# Supplementary material for: Optimization of Culture Conditions for Oxygen-Tolerant Regulatory [NiFe]-Hydrogenase Production from Ralstonia eutropha H16 in Escherichia coli
Source: Microorganisms. 2021 May 31;9(6):1195. doi: 10.3390/microorganisms9061195 (PMC8229454; doi:10.3390/microorganisms9061195)
Supplement: Supplementary file 1 [file microorganisms-09-01195-s001.zip › microorganisms-1217505-supplementary.pdf]

Fan, et al. Supplementary figures

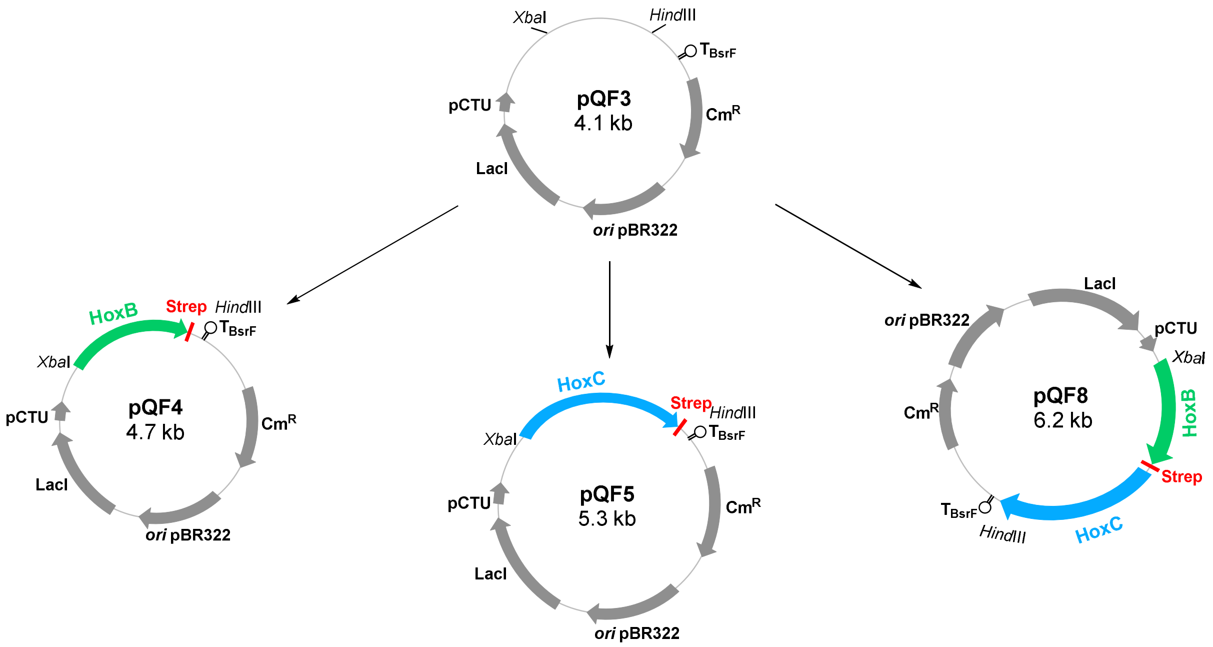

Figure S1: Plasmids used in this study

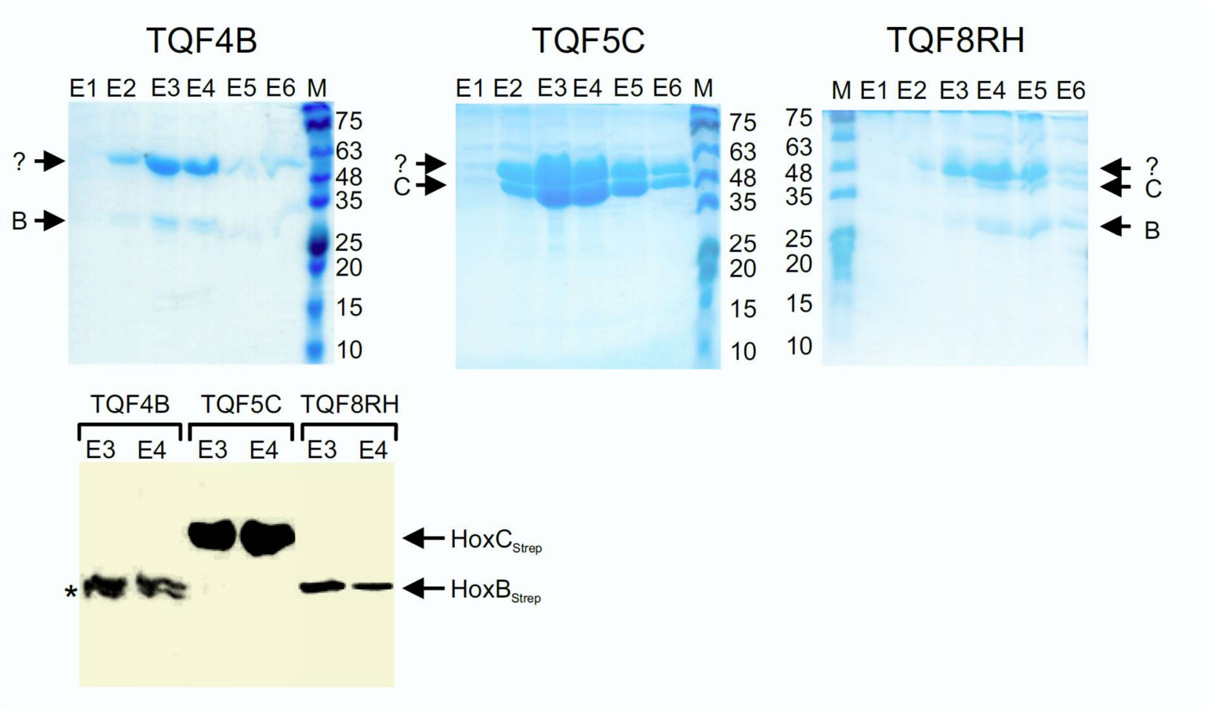

Figure S2: Analysis of Hox protein production in *E. coli* TG1

E. coli TG1 derivatives TQF4B, TQF5C or TQF8RH were cultivated in 50 ml TB medium in UYF at 37 °C. RH production was induced with 1 mM IPTG. Soluble RH was purified by affinity chromatography and subsequently analyzed by SDS-PAGE (upper panel) and Western blotting (bottom panel). 15 µL of the indicated elution fractions from the affinity purification were separated in 12 % PAA gels and either stained with colloidal Coomassie or subjected to immunoblotting using antibodies against the Strep-tag II peptide. $HoxB_{Strep}$ was purified from strain TQF4B and TQF8RH, HoxC was purified from strain TQF5C via its Strep-tag or from strain TQF8RH via co-purification with Strep-tagged HoxB. In all cases, an untagged contaminating protein is co-purified together with the Hox proteins. In preparations from TQF4B a second band with slightly lower molecular weight is detecatable indicating proteolytic digestion of HoxB. The second band is indicated by an asterisk. **B**: HoxB; **C**: HoxC; **?**: unidentified contaminating protein; M: ROTI®Mark TRICOLOR.

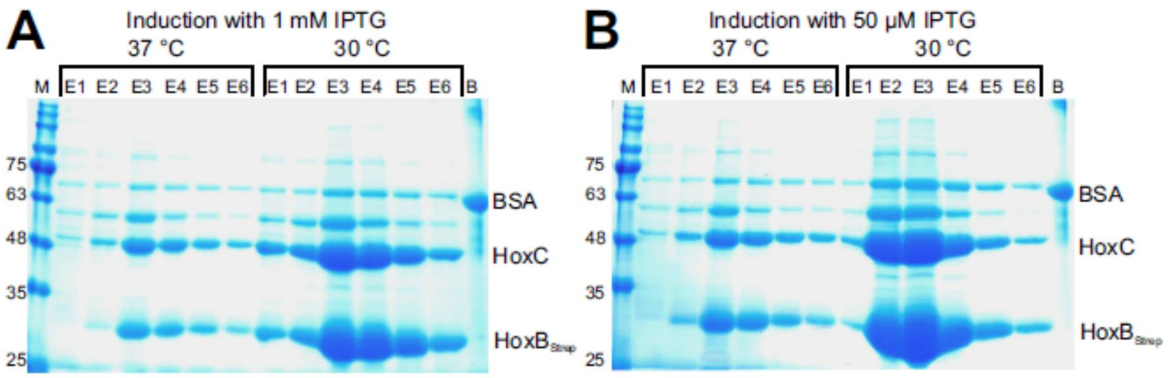

Figure S3: RH production in *E. coli* BQF8RH

E. coli BQF8RH was cultivated in 50 ml TB medium in UYF at 37 °C and 30 °C and RH production was induced with IPTG as indicated. Soluble RH was purified by affinity chromatography and subsequently analyzed by SDS-PAGE. (A) and (B) Coomassie stained gels of purified Strep-tagged HoxB and co-purified HoxC. Hox gene expression was induced with 1 mM (A) or 50 µM (B) IPTG, respectively. An amount of 15 µl of the elution fractions (E1-E6) from the affinity chromatography were loaded in each lane. ROTI®Mark TRICOLOR (Roth, Germany) (lane M) and 2 µg BSA (lane B) were used as marker

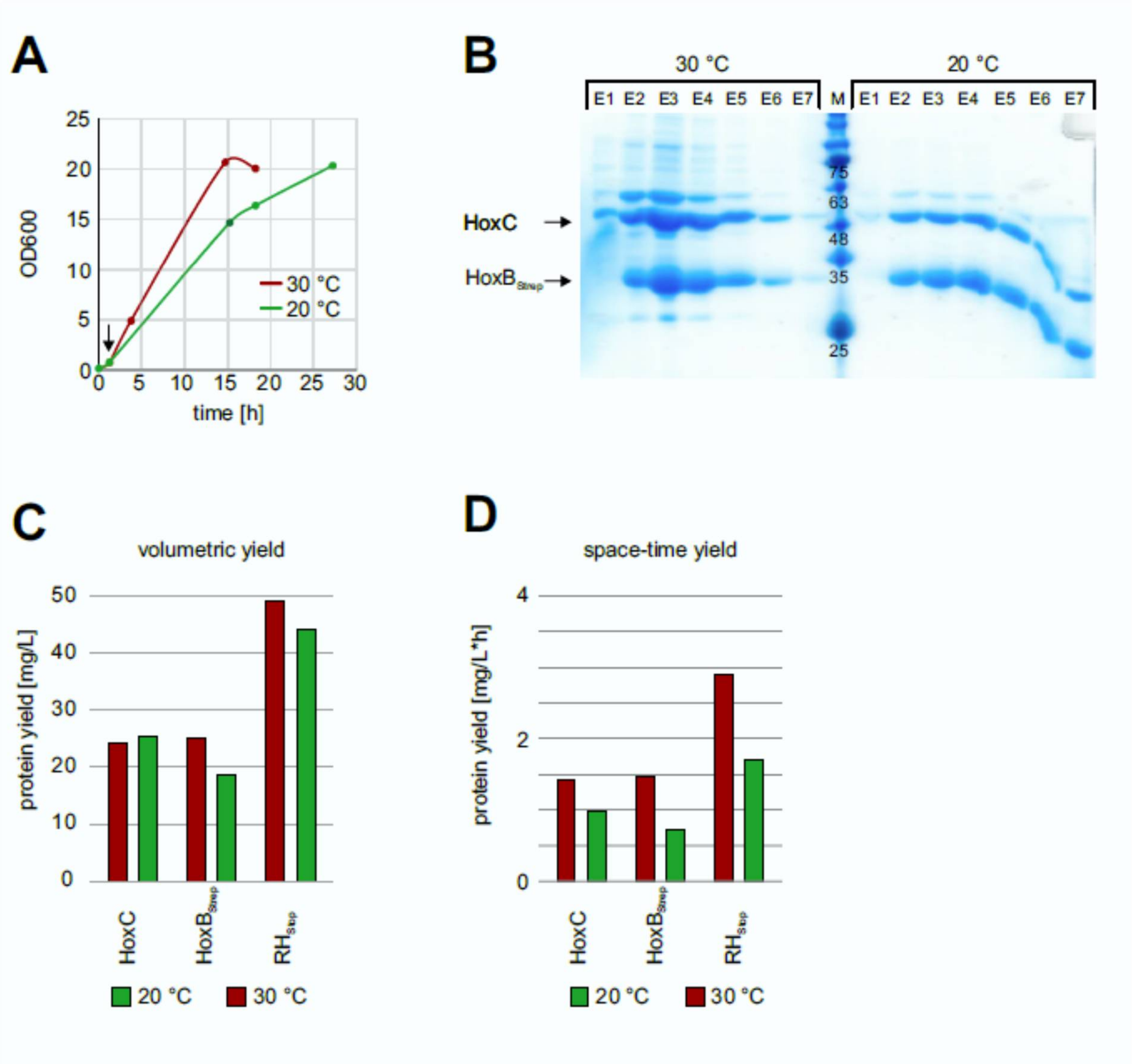

Figure S4: Comparison of RH production at 20 °C and 30 °C

E. coli strains BQF8RH was cultivated in 50 ml TB medium in UYF at 30 °C. After 75 minutes RH production was induced with 50 µM IPTG. One flask was kept for 17 h at 30 °C whereas one flask was kept at 20 °C. Due to slower growth at 20 °C cells were harvested after 26 h when an OD_{600} comparable to that of the 30 °C culture was reached. Soluble RH was purified by affinity chromatography and subsequently analyzed by SDS-PAGE. 5 µL of the indicated elution fractions from the affinity purification were separated in 12 % PAA gels and stained with colloidal Coomassie. **(A)** growth curve; **(B)** Coomassie stained gel; **(C)** comparison of volumetric yield; **(D)** comparison of space-time yield

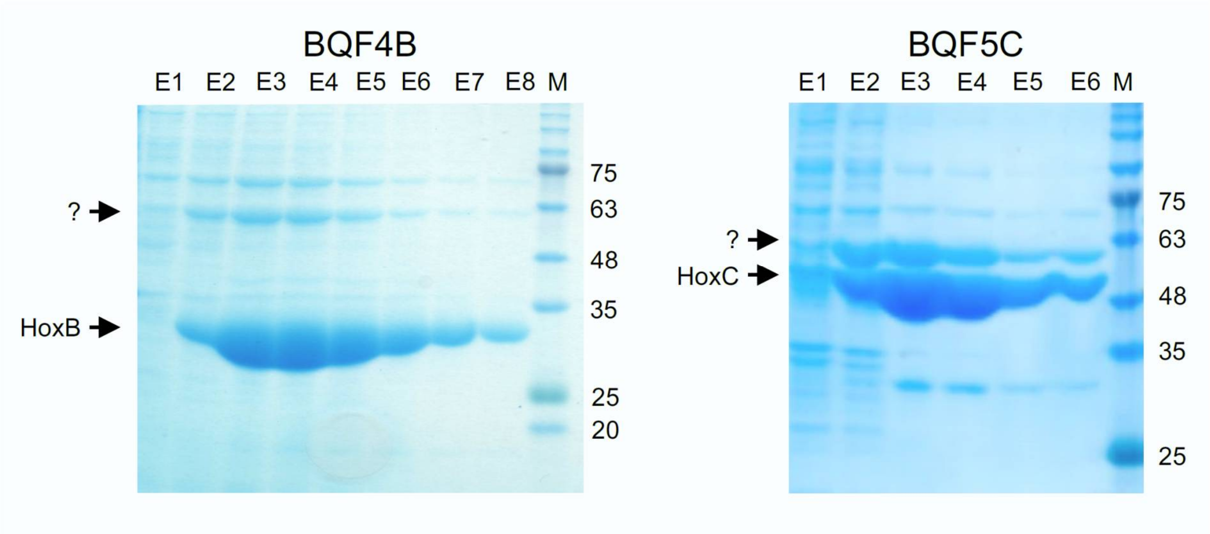

Figure S5: Production of single Hox proteins *E. coli* BL21 Gold

E. coli strains BQF4B and BQF5C were cultivated in 50 ml TB medium in UYF at 30 °C. RH production was induced with 50 µM IPTG. Soluble RH was purified by affinity chromatography and subsequently analyzed by SDS-PAGE. 15 µL of the indicated elution fractions from the affinity purification were separated in 12 % PAA gels and stained with colloidal Coomassie. $HoxB_{Strep}$ from strain BQF4B and HoxC from strain BQF5C were purified via their Strep-tag II. **?**: unidentified contaminating protein; M: ROTI®Mark TRICOLOR.

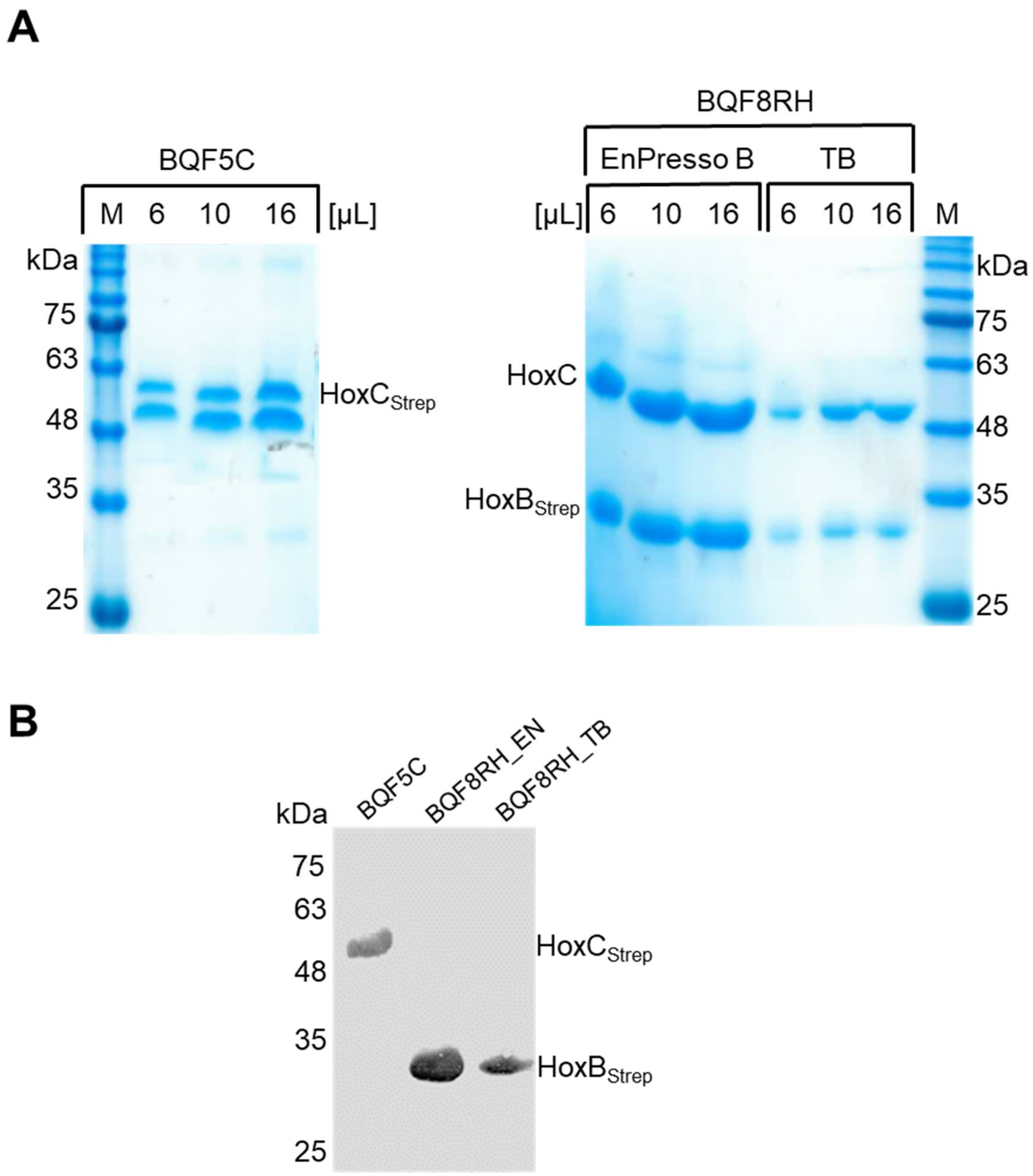

Figure S6: SDS-PAGE analysis of purified HoxC and RH used for spectroscopic characterization

E. coli strains BQF5C and BQF8RH were cultivated in 50 ml TB medium or boosted EnPresso B medium in UYF at 30 °C, 250 rpm. RH production was induced with 50 µM IPTG. Soluble RH was purified by affinity chromatography. Subsequently, the elution fractions E2-E6 were pooled and concentrated by ultrafiltration using Amicon filter (30 kDa) ready for activity measurement and spectroscopic characterization. The concentrated sample was diluted to approx. 0.5-1.0 mg/L measured by NanoDrop (A280 nm) and subsequently appropriate diluted sample volumes were separated in 12 % PAA gels and stained with colloidal Coomassie (**A**) and analyzed by Western blotting with antibodies against Strep-tag (**B**). M: ROTI®Mark TRICOLOR.

Fan, et al. Supplementary tables

Table S1: Strains used in this study

Strain	Genotyp	Reference
E. coli TG1	*E. coli K-12 glnV44 thi-1 Δ(lac-proAB) Δ(mcrB-hsdSM)5(r_K–m_K–) F' [traD36 proAB$^+$ lacI^q lacZΔM15]*	Baer *et al.*, 1984
E. coli BL21 Gold	*E. coli B F$^–$ompT hsdS(r_B– m_B–) dcm$^+$ Tet^R gal endA The*	Agilent, Waldbronn
E. coli TQF4B	as TG1 with plasmid pQF4	This work
E. coli TQF5C	as TG1 with plasmid pQF5	This work
E. coli TQF8RH	as TG1 with plasmid pQF8	This work
E. coli BQF4B	as BL21 Gold with plasmid pQF4	This work
E. coli BQF5C	as BL21 Gold with plasmid pQF5	This work
E. coli BQF8RH	as BL21 Gold with plasmid pQF8	This work

Table S2: Plasmids used in this study

Plasmid	Description	Reference
pCTUT7	*E. coli* cloning vector, Cm^R, P_{lac}, MCS	Kraft *et al*. 2007
pCH594	*E. coli* cloning vector, Amp^R, P_{SH}, *hoxB, hoxC, hoxJ*	Kleihues *et al*. 2000
pQF1	as pCTUT7 with deletion of unique BstBI site	This work
pQF3	as pQF1 with integration of *bsrF* transcription terminator	This work
pQF4	as pQF3 with integration of *hoxB*	This work
pQF5	as pQF3 with integration of *hoxC*	This work
pQF8	as pQF3 with integration of *hoxBC*	This work

Table S3: Oligonucleotides used in this study

Name	Sequence (starting with 5' end)	purpose
MG0034	CGT CGA CTC GAG CTC GCT GCA GA	C, pQF1
MG0035	CGT CTG CAG CGA GCT CGA GTC GA	C, pQF1
MG0038	AGT **TCT AGA** TGG CTT GGA GGA GAA ATG AAC GCG CCT GTA TGT	C, pQF4
MG0039	GTT TGG TGG AAG GGG TGG CCG GGC TCC TCA AAG CCG GGT TCA GTG CAA	C, pQF4
MG0040	ATC **AAG CTT** A**TT TTT CGA ACT GCG GGT GGC TCC A**AG CAG AGG GTG TTT GGT GGA AGG GGT GG	C, pQF4
MG0041	CAG **TTC GAA** AAA TAG GAG GCG AGC ATG GAA CGT TTG	C, pQF8
MG0042	ATC **AAG CTT** TCA ATG CAC GGT GCA CAC CAT G	C, pQF8
MG0043	ATG **TCT AGA** AAT AGG AGG CGA GCA TGG AAC	C, pQF5
MG0044	ACT **AAG CTT** A**TT TCT CAA ACT GCG GGT GGC TCC A**AG CAG AAT GCA CGG TGC ACA CCA TGC AG	C, pQF5
MG0046	CGA ACG TGG CGA GAA AGG AA	S
MG0051	AGC TAC TAG TAG ATC TAA AAA GGC GTT TGG CTA AGG CAA ACG CCT TTT TAA GCT TG	C, pQF3
MG0052	GAT CCA AGC TTA AAA AGG CGT TTG CCT TAG CCA AAC GCC TTT TTA GAT CTA CTA GT	C, pQF3
M13-24R	CGG ATA ACA ATT TCA CAC AGG	S

–

S, sequencing; C, construction of plasmid followed by plasmid designation; Strep-tag sequence is highlighted in red; restriction sites are underlined
